# Continuous surveillance of potentially zoonotic avian pathogens detects contemporaneous occurrence of highly pathogenic avian influenza viruses (HPAIV H5) and flaviviruses (USUV, WNV) in several wild and captive birds

**DOI:** 10.1080/22221751.2023.2231561

**Published:** 2023-07-11

**Authors:** Anne Günther, Anne Pohlmann, Anja Globig, Ute Ziegler, Sten Calvelage, Markus Keller, Dominik Fischer, Christoph Staubach, Martin H. Groschup, Timm Harder, Martin Beer

**Affiliations:** a Institute of Diagnostic Virology, Friedrich-Loeffler-Institut, Federal Research Institute for Animal Health, Greifswald-Insel Riems, Germany; b Institute of International Animal Health/One Health, Friedrich-Loeffler-Institut, Federal Research Institute for Animal Health, Greifswald-Insel Riems, Germany; c Institute of Novel and Emerging Infectious Diseases, Friedrich-Loeffler-Institut, Federal Research Institute for Animal Health, Greifswald-Insel Riems, Germany; d Der Gruene Zoo Wuppertal, Wuppertal, Germany; e Institute of Epidemiology, Friedrich-Loeffler-Institut, Federal Research Institute for Animal Health, Greifswald-Insel Riems, Germany

**Keywords:** *Orthomyxoviridae*, *flaviviridae*, raptors, disease surveillance, host species, HPAIV H5, USUV, WNV

## Abstract

Three avian viral pathogens circulate in Germany with particular importance for animal disease surveillance due to their zoonotic potential, their impact on wild bird populations and/or poultry farms: Highly pathogenic (HP) avian influenza virus (AIV) of subtype H5 (HPAIV H5), Usutu virus (USUV), and West Nile virus (WNV). Whereas HPAIV H5 has been mainly related to epizootic outbreaks in winter, the arthropod-borne viruses USUV and WNV have been detected more frequently during summer months corresponding to peak mosquito activity. Since 2021, tendencies of a potentially year-round, i.e. enzootic, status of HPAIV in Germany have raised concerns that *Orthomyxoviruses* (AIV) and *Flaviviruses* (USUV, WNV) may not only circulate in the same region, but also at the same time and in the same avian host range. In search of a host species group suitable for a combined surveillance approach for all mentioned pathogens, we retrospectively screened and summarized case reports, mainly provided by the respective German National Reference Laboratories (NRLs) from 2006 to 2021. Our dataset revealed an overlap of reported infections among nine avian genera. We identified raptors as a particularly affected host group, as the genera *Accipiter*, *Bubo*, *Buteo*, *Falco*, and *Strix* represented five of the nine genera, and highlighted their role in passive surveillance. This study may provide a basis for broader, pan-European studies that could deepen our understanding of reservoir and vector species, as HPAIV, USUV, and WNV are expected to further become established and/or spread in Europe in the future and thus improved surveillance measures are of high importance.

## Introduction

In recent years, various epizootics have drawn attention to the increasing spread of animal pathogens, circulating between wildlife, livestock, and pet animals and being potentially spilled-over to humans. Three zoonotic viruses are especially relevant in wild bird populations that permanently reside, breed, and winter within or migrate through Germany: highly pathogenic (HP) avian influenza viruses (AIV) of subtype H5, and the flaviviruses Usutu virus (USUV) and West Nile virus (WNV). Until now, their spatio-temporal occurrence and host range in Germany has not been investigated in a combined retrospective study in order to identify any potential overlaps in the emergence and/or maintenance of these viruses.

The first cases of HPAIV H5N1 in Germany in winter 2006 were caused by incursions of the Asian H5 A/Goose/Guangdong/1/1996 (gs/GD) lineage. These viruses belonged genetically to clade 2.2 of the gs/GD lineage, whereas since 2014 clade 2.3.4.4 viruses are dominating [1]. Until 2021, temporal and spatial patterns of gs/GD HPAI virus emergence were correlated with the presence of migrating or resting wild waterfowl in Germany. The threat of incursion into poultry flocks has increased, and wild bird populations themselves have suffered severely during and amidst epizootic events [2–8]. Some waterfowl such as various dabbling duck species, which are a long-known reservoir for low pathogenic AIV, may not even show clinical signs after infection with HPAIV H5 [9,10]. The considerable contagiousness of HPAIV, the orofecal transmission route, and their substantial tenacity in the environment [11] in combination with the seasonal behaviour of birds, in particular mixed-species flocking at resting areas during migration or aggregating in winter, supports the interspecies distribution.

USUV and WNV belong to the group of arthropod-borne (ARBO) viruses, as they are mainly transmitted in an enzootic cycle between wild birds and ornithophilic mosquito species (especially *Culex sp.*) [12,13]. Thus, the virus activity is dependent on the susceptibility of the local bird species, but also on vector competence and availability associated to environmental conditions. The first evidence for USUV in Germany was detected in a *Culex pipiens pipiens* pool, trapped in summer 2010 in southwest Germany in the context of a mosquito-monitoring programme [14]. A regional outbreak in a local passerine bird population was described one year later, followed by regular detections across the country since 2018 [15,16] and a meanwhile enzootic status [17–19]. Several studies confirmed the vector competence of local arthropod species in Germany for USUV and WNV [12,13,20].

In contrast, WNV reached Germany not before 2018. In the following years, enzootic outbreaks in wild bird populations developed in the eastern part of the country based on various introduction events of WNV strains of Eastern European origin. Again, those outbreak events mostly correlated with main mosquito activity in summer months [12,21]. Although WNV was not confirmed all over Germany, it became enzootic in so called “hotspot areas” in Berlin and Central-Germany [16,18,20,22–26].

Recently, concerns have been raised that HPAIV H5 (clade 2.3.4.4b) might have established an enzootic status in Europe since 2021, as productive wild bird infections and outbreaks in poultry holdings are now occurring year-round including the summer months [27]. In this regard, spatio-temporal co-circulation of HPAIV H5, USUV, and WNV in German wild bird populations is to be anticipated in the near future.

We therefore evaluated this potentially year-round threat by conducting a data- and literature-based study to identify susceptible avian species for these pathogens in Germany. Our aim was to identify a possible overlap of species, genera, or avian groups that might be utilized as future indicators for the circulation of HPAIV H5, USUV, and WNV in Germany to support combined surveillance approaches of orthomyxoviruses and flaviviruses with zoonotic potential.

## Material and methods

Reports and databases issued by the German Reference Laboratories (NRLs) for WNV and AIV, based at the Friedrich-Loeffler-Institut (FLI), Isle of Riems, were screened systematically to gain information on affected bird species. According to the first confirmation of each virus in Germany, the dataset included cases from 2006–2007, 2009, 2014–2017 and 2020–2021 (for infections with HPAIV H5), 2011–2021 (for infections with USUV) and 2018–2021 (for infections with WNV). The majority of these retrospectively examined cases were already part of monitoring programmes on the respective pathogens in Germany within the last years [1,3,5–8,15–19,22–25,28–33].

Information on affected hosts by at least one of the three viruses was collected, if the following criteria applied:

### Sample origin and time

The sample had to be collected in Germany. The previous diagnostics of the reported cases were mainly based on swab samples or organ material for HPAIV H5 confirmation and organ material or blood clot for flavivirus detection (see original publications). We considered reported cases in wild birds and included reports on captive wild bird species, including zoological institutions and private facilities. Since it was not possible in all cases to ascertain the exact date of sampling or the time when a dead animal was found, we applied the date when the animal was found dead, the date when the animal was sampled or, if none of the former was available, the date when the sample arrived at the respective NRLs.

### Animal disease

We screened the data for reports on HPAIV H5, USUV, and WNV infections. For AIV, we focused on infections with HP subtype H5, as these strains were the dominating ones in Europe since 2006 and are known to harbour zoonotic potential [1,34]. Our dataset covers different time periods related to the virus’ first detection in Germany (HPAIV since 2006, USUV since 2011, and WNV since 2018) and therefore, comprises different genetic strains as investigated by prior independent studies that include sequences and corresponding metadata.

### Infection status

We included individual birds that tested positive for viral RNA by RT-qPCRs as described by Michel et al. [16] and Hassan et al. [35], regardless of any clinical signs, pathological lesions, or death. A case within our dataset does not necessarily indicate the death or euthanasia of the affected animal, nor if the sample stands for a single individual or was one tested bird representing a group of birds at one sampling site. High detection rates are achieved in passive surveillance approaches based on swab-sampled carcasses or organ material, thus, the majority of samples were most likely from deceased birds. However, for the analysed blood clot, the status of the individual remains unclear.

### Species

We focused on avian host species and did not include reports in humans, mammalian, or arthropod-vector species. A report was excluded, if the taxonomy has not been indicated and the individual could not be classified at least on the taxonomic level of orders. Beside wild birds, we included cases in various captive birds, except domestic poultry species, in order to broaden the spectrum of contemplable host species. In this regard, the following species and species groups were considered poultry and were thus not included in the study: domestic fowl/chicken, domestic goose, domestic duck, quail, and turkey. Poultry is rarely known to perish following flavivirus infections. Therefore, poultry species are unlikely to be suitable targets for passive surveillance approaches, nor suitable reservoir hosts for all three avian pathogens mentioned above. Information on taxonomic identification was summarized with the most precise biological characterization (lowest taxonomic level) given: order, family, genus, and species. If it was not possible to assign the exact species name, the next higher, possible taxonomic level was applied.

## Results

### Identification of host species with overlapping occurrence for orthomyxo- and flaviviruses

**Figure 1.**
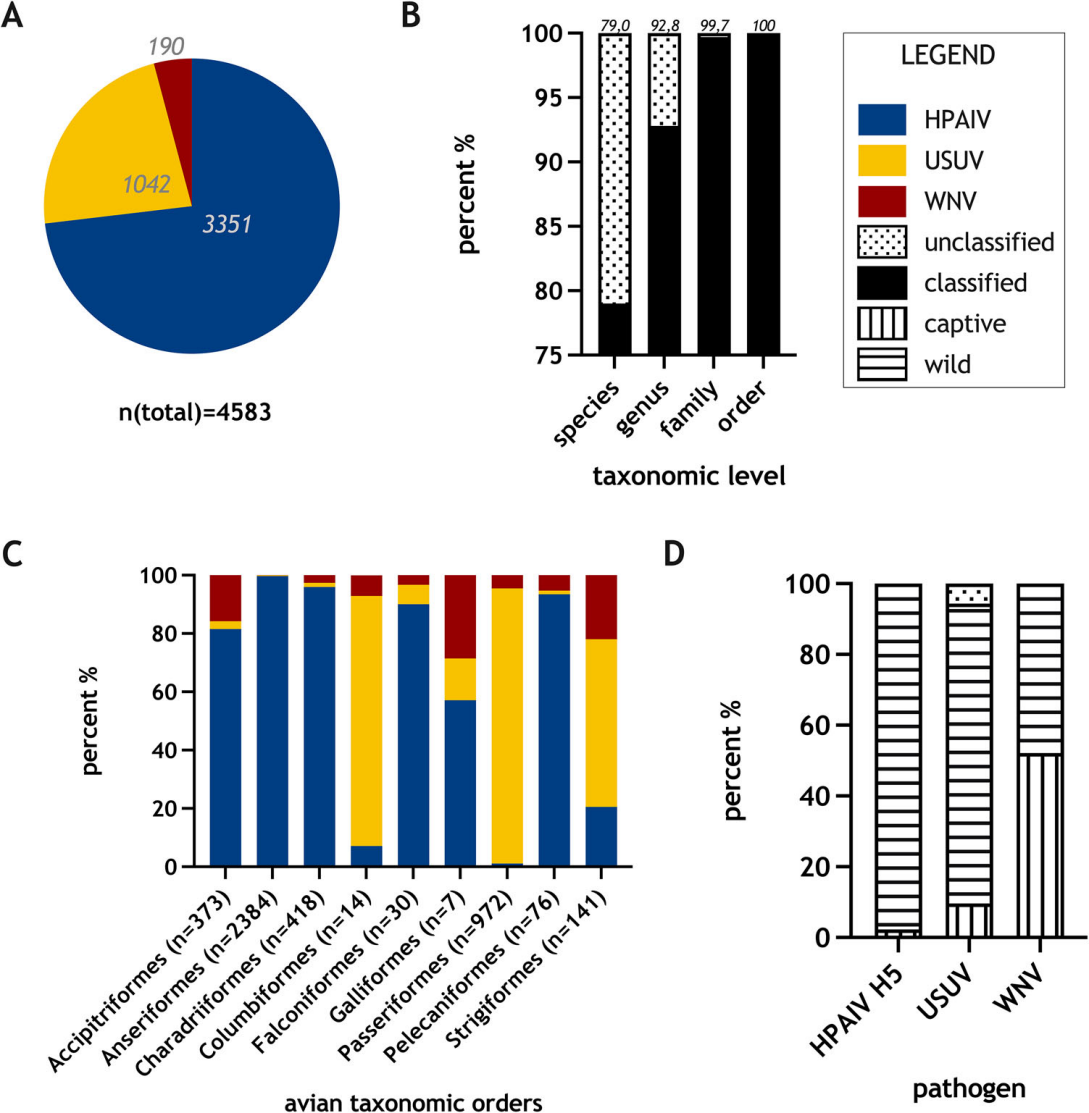
Overview of test results and avian taxa, which were tested positive by RT-qPCR for highly pathogenic avian influenza virus of subtype H5 (HPAIV H5), Usutu virus (USUV), and West Nile virus (WNV) in Germany from 2006 to 2021. (A) Number of positive RT-qPCR test for HPAIV H5, USUV, and WNV; (B) Eligibility to biological classification (species, genus, family, and order) displayed as percentage from the total number of samples; (C) Distribution of positive test results for HPAIV H5, USUV, and WNV in nine avian orders, reported for infections with all mentioned pathogens; (D) Origin of birds tested positive for HPAIV H5, USUV, and WNV, distinguishing between captive and wild.

In total, 4583 cases of avian individuals, infected by HPAIV H5, USUV, or WNV have been identified (Supplementary Table S1), whereby 73.1% refer to AIV (*n* = 3351), 22.7% to USUV (*n* = 1042), and 4.2% to WNV infections (*n* = 190; Figure 1(A)). Cases within our dataset were not usually screened for all three pathogens. For seven individuals, a co-infection with both flaviviruses was described. These seven cases were included twice in the list of detected infections, listed once for USUV and once for WNV, and marked with an asterisk in the column “co-infection” in Supplementary Table S1 [18,23]. Co-infections with flaviviruses occurred in 2018, 2019, and 2020 among captive and wild individuals of *Accipitriformes*, *Anseriformes*, *Charadriiformes*, *Passeriformes*, and *Strigiformes.* Thus, they represented 1.1% of all USUV and 3.7% of all WNV cases since both pathogens co-existed in Germany in 2018. No individual bird was confirmed to harbour a co-infection with one or both Flaviviruses together with HPAIV H5.

Regarding biological classification/taxonomy, in 3622 out of 4583 cases (79.0%), the avian species was indicated and 136 different avian species were covered in total. However, in 961 reports (21.0%) no precise species was indicated (Figure 1(B)). The less specific the biological classification (higher taxonomic level), the more cases were classified.

Figure 1(C) shows nine avian orders in which infections with all three pathogens were detected. HPAIV H5 infections were reported mainly in *Accipitriformes*, *Anseriformes*, *Charadriiformes*, *Falconiformes*, *Galliformes*, and *Pelecaniformes*. USUV infections prevailed in *Columbiformes*, *Passeriformes*, and *Strigiformes*. WNV infections represented the minority of cases in all bird orders. Highest numbers of WNV infections were identified in *Accipitriformes*, *Strigiformes*, and *Galliformes*, although in the latter order only a total number of seven cases was reported.

While for HPAIV H5 and USUV infections, cases were mainly reported in wild birds, WNV infections were identified in captive and free-ranging birds nearly equally distributed (Figure 1(D)). As indicated above, cases in poultry species have not been considered here.

### Pronounced overlapping incidence for orthomyxoviruses and flaviviruses in raptors and scavengers

**Figure 2.**
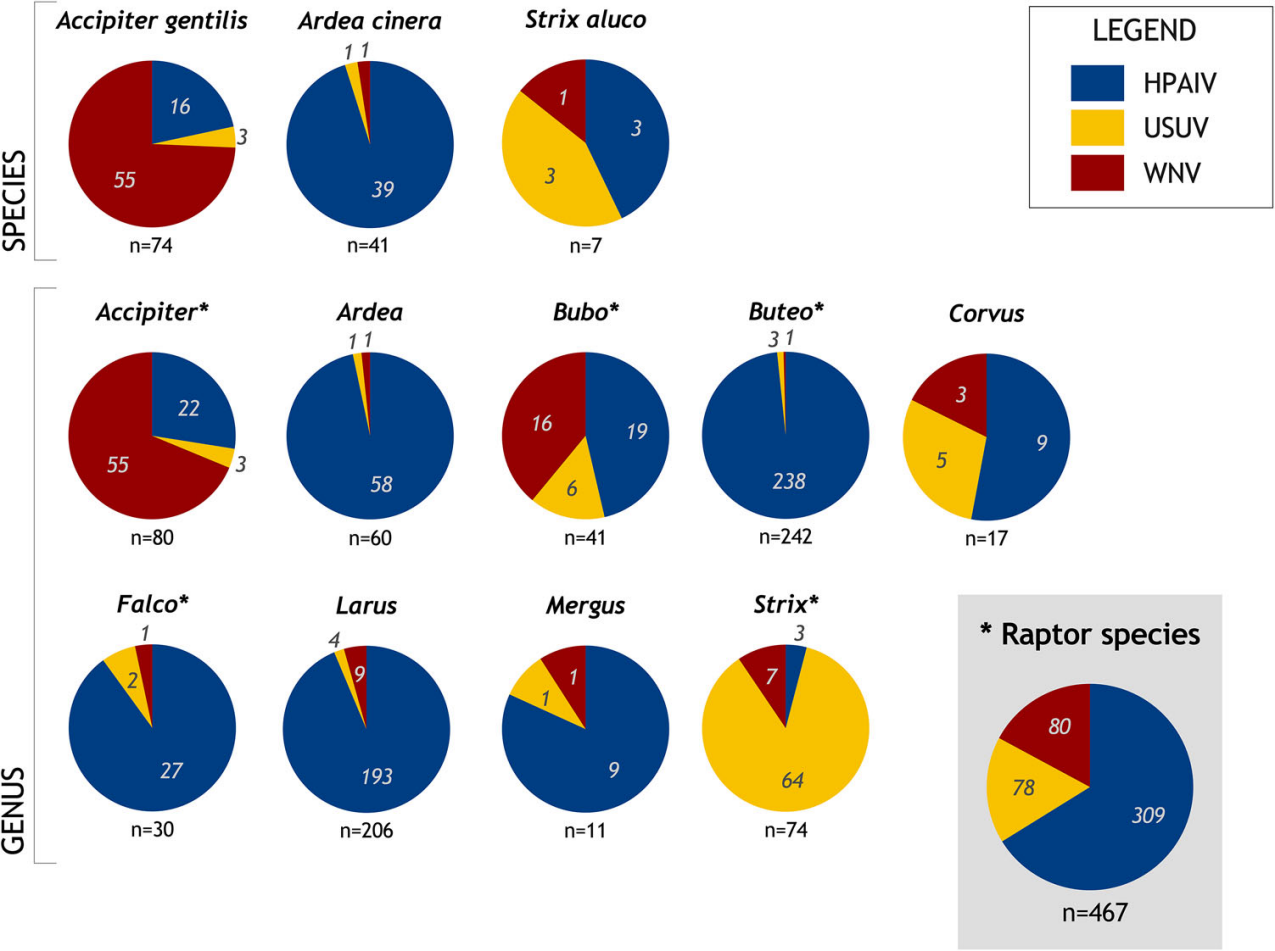
Distribution of pathogen detection in species, genera, and groups showing infections with three viruses (highly pathogenic avian influenza virus of subtype H5 (HPAIV H5), Usutu virus (USUV), and West Nile virus (WNV)) detected from 2006 to 2021. Genera belonging to the group of raptors are marked with an asterisk.

The comparison of the affected hosts revealed that all pathogens were detected at least once in three avian species (Figure 2): Northern goshawk (*Accipiter gentilis*), tawny owl (*Strix aluco*), and grey heron (*Ardea cinerea*). Therefore, those species represented an overlap in general predisposition. Reports for northern goshawks (*n* = 74) mainly referred to WNV infections (HPAIV H5 21.6%, USUV 4.1%, WNV 74.3%), whereas in tawny owls (*n* = 7) USUV and HPAIV H5 infections were described more often than WNV infections (HPAIV H5 *n* = 3, USUV *n* = 3, WNV *n* = 1). Grey herons (*n* = 41) were found only once positive for USUV or WNV, while HPAIV H5 infections were confirmed in the majority of the reports (HPAIV H5 95.1%, USUV 2.4%, WNV 2.4%).

The comparison on higher taxonomic levels gave a similar picture (Figure 2): In the genus *Accipiter spp*., WNV infections dominated. In *Bubo spp*., WNV infections were reported almost as frequently as HPAIV H5 infections. HPAIV H5 infections occurred mainly in the genera *Ardea spp.*, *Buteo spp.*, *Corvus spp.*, *Falco spp.*, *Larus spp*., and *Mergus spp*. Only for the genus *Strix spp*. USUV infections were the most common reported infection.

A summary of cases in the avian host genera *Accipiter*, *Bubo*, *Buteo*, *Falco*, and *Strix* is displayed in Figure 2 under the term “raptor species”.

### Temporally overlapping co-circulation of HPAIV H5 and WNV in wild birds in Germany

**Figure 3.**
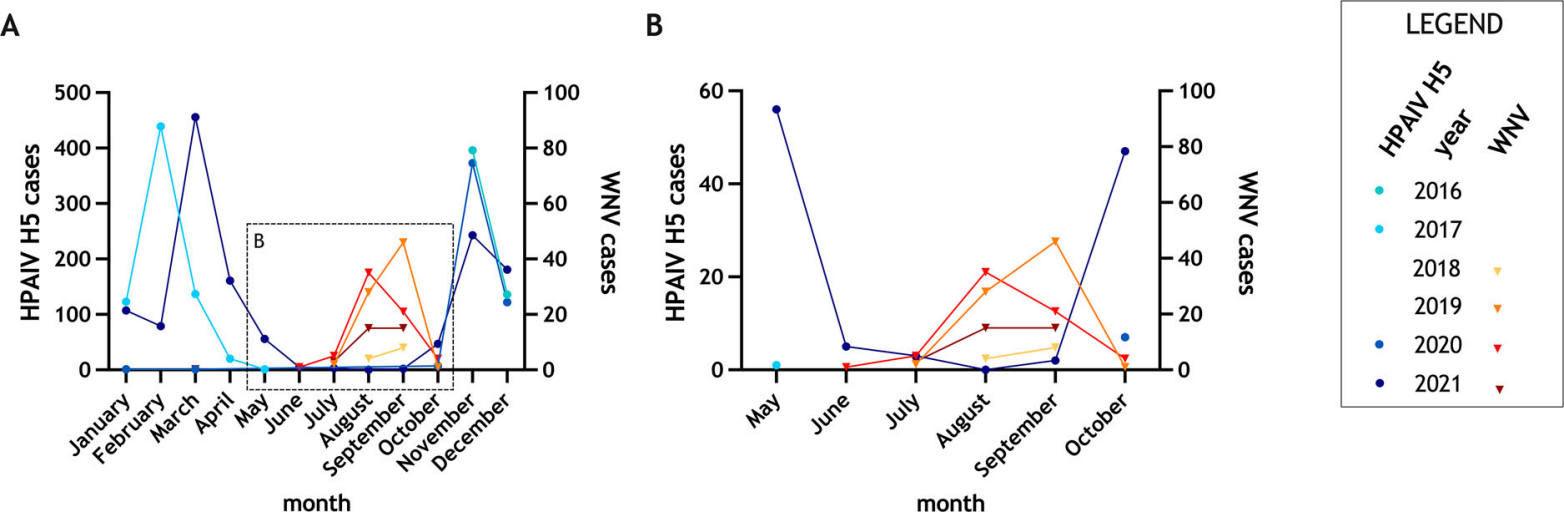
Time of highly pathogenic avian influenza of subtype H5 (HPAIV H5) and West Nile virus (WNV) infections reported in birds, stratified by month, displayed for the years 2016–2021. (A) Annual view, (B) Focus on summer and autumn months (May to October).

For case reports of HPAIV H5 and WNV infections from 2016 to 2021, the date (sampling date or the date when the post-mortally tested individual was found) was associated. Where this information was not available, the arrival date of the sample at the NRLs at FLI, was considered instead. Figure 3 displays the temporal dynamics for both pathogens (see also Supplementary Table S2). For the USUV subset, this information was given only sporadically and therefore USUV data were not included here.

In general, until 2021 HPAIV H5 activity occurred mainly in autumn and late spring. However, HPAIV outbreaks in 2021 no longer revealed the seasonal pattern as in previous years and high numbers of wild bird cases were also reported during the summer.

WNV infections were reported from June to October except for one case in March. That case was confirmed in March 2021 for a WNV-positive tested Jandaya parakeet (*Aratinga jandaya*) kept in an aviary in a zoo in Berlin. It is very likely that this positive test was the result of a chronic infection, as viral genome detection was only possible in the kidney of the bird, but not in other organs.

Months with overlapping activities of WNV and HPAIV H5 were June, July, September, and October in the present study. Individuals, which were tested positive for WNV and also for USUV at the same time, were reported between the end of August and the beginning of September.

## Discussion

Demands for disease monitoring in wild bird populations focusing on potentially zoonotic viruses increased recently, and in Germany, HPAIV, USUV, and WNV became the most relevant ones. Combining monitoring efforts might enable (i) broader surveillance on virus activity in general, (ii) simplified and resource-sparing approaches by targeting suitable indicator species/groups, and to (iii) optimize risk assessments for spill-over events to humans.

Although excluded in this first attempt, the poultry sector represents a human-bird interface (e.g. farmers, veterinarians, consumers) and there is a risk of pathogen spill-back from poultry holdings into wild bird populations, particularly for HPAIV H5 (e.g. due to improper biosafety measures or in free-ranging flocks). For Arboviruses, the role of chicken, duck, and goose as reservoir or carrier is minor. These species have been discussed as indicator species for flavivirus circulation, but only on the basis of serological findings [36]. In this context, the absence of severe viremia, e.g. after WNV infections, hampers analyses on the genetic background of the respective virus strain, that could be obtained by RT-qPCR screening and sequencing within passive monitoring approaches in wild or zoo birds.

The majority of case reports were collected for infections with HPAIV H5, followed by USUV and comparably few cases of WNV (Figure 1(A)). This distribution might reflect the respective time period after the pathogens’ first introduction into German wild bird populations: HPAIV H5 (clade 2.2) in 2006 [3] and clade 2.3.4.4 in 2014 [2], USUV (Europe 3) in 2011 [15], and WNV (lineage 2) in 2018 [25]. Moreover, the higher contagiousness and the direct transmission cycle of HPAIV H5 compared to the less effective, slower and primarily vector-dependent transmission of WNV and USUV might have led to this distribution.

About 79% of all reports could be assigned to the lowest taxonomic level of the precise avian species (Figure 1(B)). Multiple uses of generalizing terms such as buzzard, wild duck, thrush, or raptor prevented the identification of the exact avian species in 21% of the cases and only allowed the more general taxonomic classification up to the level of orders. Therefore, we emphasize the importance of accurate species identification during sampling to enable a precise data assessment. Animal photographs accompanied with samples and reports might help to determine the species retrospectively. Furthermore, e.g. (official) veterinarians in charge could be specifically trained for species identification or be provided with survey sheets. Molecular determination of host species, e.g. by utilizing DNA barcodes [37], might be done when a taxonomic classification is not possible based on morphology. Moreover, all data must be reported to the databases in a complete and detailed manner, and databases should be encouraged or forced to use appropriate species catalogues minimizing typing and reporting errors. Missing data on the exact species impedes the idea of picking certain species for monitoring attempts, not only in a combined approach. However, any knowledge about the host taxonomy might help to understand the spread of viral pathogens that is influenced not only by characteristics of the viral entity but also by the species-specific characteristics of the host. One example might be the migrations of waterfowl and the association of this behaviour to the spread of HPAIV H5 [2].

Although we figured out avian groups affected by all three pathogens, none of the numerically abundant species/genera showed an evenly distributed pattern of the pathogens (Figure 1(C) and Figure 2). Choosing a single of those species/genera, e.g. representative for one pathogen, for monitoring, could harbour the risk of overlooking other pathogens.

Common to all but one overlapping genera (except *Mergus*) is the scavenging and/or hunting behaviour of the birds. Therefore, we regrouped subsets according to characteristics of host species, instead of only their taxonomic attribution: Datasets for five genera of birds of prey (*Accipiter*, *Buteo*, and *Falco*) and owls (*Bubo* and *Strix*) were summarized under the term raptors. Thus, a slightly more harmonized distribution pattern became apparent for infections with WNV, USUV, or HPAIV H5. The position as predator at the end of the food chain, feeding on carrion or hunting infected and therefore potentially weakened avian prey species results in a certain risk of increased exposure to pathogens and thus hunts towards their suitability as indicators.

Various studies describe raptor species as susceptible for orthomyxoviruses [8,38,39] or flaviviruses [22,40–42], mainly transmitted by the alimentary route. Medium-sized and larger raptors seem to be especially attractive to mosquitoes [43]. Some residential raptor species in Germany may be categorized as medium-sized and might attract public attention when found weakened or dead. Smaller birds such as most songbird species are more easily overlooked, except in the case of mass mortalities in a circumscribed region. Moreover, small-sized birds are often caught, especially when sick, and their carcasses are faster removed by raptors or by mammalian predators such as cats, foxes, martens, or racoons. In addition, the popularity of raptor species might support rescue attempts by citizens when they observe, for example, neurological signs of disease as described for infections with WNV or USUV but also with HPAIV (see Supplementary Table S3) and, thus, helps retrieving cases [8,22,39,41,42,44–55]. At the same time, the described circumstances could lead to a pre-selection of species of which samples will ultimately be tested within routine diagnostics. To that effect, it can only be speculated about the existence of further species (groups) possibly representing an overlap in general predisposition, but not being tested.

Although there was no indication for a single suitable species, our data suggest us to recommend to always test raptor species (or scavenging species) for all mentioned pathogens, especially in passive disease monitoring programmes. This synergizes with the relevance of raptors for ecotoxicological monitoring approaches (monitoring pesticides, rodenticides, and heavy metals). This comprises European programmes as ERBFacility, EuRapMon [56], and MEROS [57,58]. In order to optimize efficacious use of raptor samples, such monitoring programmes should be combined in future, if biosafety aspects are not violated.

After its introduction in 2018, infections with WNV lineage 2 were reported mainly during summer months – whereas HPAIV H5 outbreaks (clade 2.3.4.4b) were associated with epizootics in the winter semester (Figure 3). Since 2020 this situation has changed, as HPAIV-H5-cases of various species were reported in late spring and even during summer [27,59] culminating in HPAI-associated mass mortalities of colony breeding sea birds in Europe in summer of 2022 [60]. The tendency of HPAIV activity continuing in late spring and summer months was recognized in other European countries as well and exacerbated the concern of HPAIV H5 becoming enzootic in Europe [27]. The years 2020/21 therefore represented the first cycles of overlapping outbreak scenarios of HPAIV H5 and WNV in Germany, as WNV was detected from June to October 2020 and July to September 2021. Mild winters and the ability of e.g. WNV-positive vectors to overwinter might also extend the time period of flavivirus occurrence in wild bird populations in the future [26].

The included co-infections with USUV and WNV in avian species were described as the first cases for Europe by Santos et al. [18] and later on by Ziegler et al. [23] (Supplementary Table S1). Given four years of co-existing WNV and USUV circulation, such co-infections seem to occur quite infrequently, possibly due to cross-protective immunity or interference effects amongst flaviviruses. However, no reports on interference between Flaviviruses and Orthomyxoviruses in birds (wild, captive, or poultry species) and co-infections with HPAIV H5 and WNV have been published. Such a scenario seems unlikely, as an infection with one of these pathogens ends lethally usually for the majority of avian hosts, except waterfowl species perhaps. Furthermore, once a pathogen is detected in a deceased bird, it is often considered as the causative agent of death and no further assays targeting other diseases are conducted. To investigate Flavivirus–Orthomyxovirus co-infections in birds, positive cases would have to be re-tested for the corresponding pathogen.

Surveillance measures at a national level could be improved if continued in the context of transboundary approaches, e.g. global climate change may affect bird migration routes or behaviour and, thus, potentially disease infection patterns.

## Conclusion

This study on infections with HPAIV H5, USUV, and WNV among wild and captive birds in Germany succeeded in identifying a spatio-temporal overlap of affected host species or genera and pathogen occurrence. Although it could not be shown for a single bird species/genus, our data particularly highlight the role of raptors for combined passive surveillance of orthomyxoviruses and flaviviruses.

Due to the increasing and partially overlapping infection pressure of wild birds particularly by HPAIV H5 and WNV, orchestrated European-wide studies generating transnational datasets would allow a more comprehensive view on affected bird species across their habitats in the European geographic range. Such an approach might reveal further insights into reservoir and carrier species. These pathogen-targeting studies could be combined with existing ecotoxicological studies for synergistic effects aiming at important key wild bird species such as apex-predators. Given the zoonotic potential of both HPAIV H5 and WNV interdisciplinary collaboration among infectologists, environmental toxicologists, and ornithologists in a One Health frame is highly recommended. Parallel monitoring of vectors, humans, and susceptible animal hosts increases the likelihood, effectiveness, and timeliness of pathogen detection and the validity of pathogen distribution patterns offering various advantages for veterinary and human medicine.

## Supplementary Material

Supplemental Material

Supplemental Material
