# Supplementary figures and images for: Continuous surveillance of potentially zoonotic avian pathogens detects contemporaneous occurrence of highly pathogenic avian influenza viruses (HPAIV H5) and flaviviruses (USUV, WNV) in several wild and captive birds
Source: Emerg Microbes Infect. 2023 Jul 11;12(2):2231561. doi: 10.1080/22221751.2023.2231561 (PMC10337501; doi:10.1080/22221751.2023.2231561)

2016

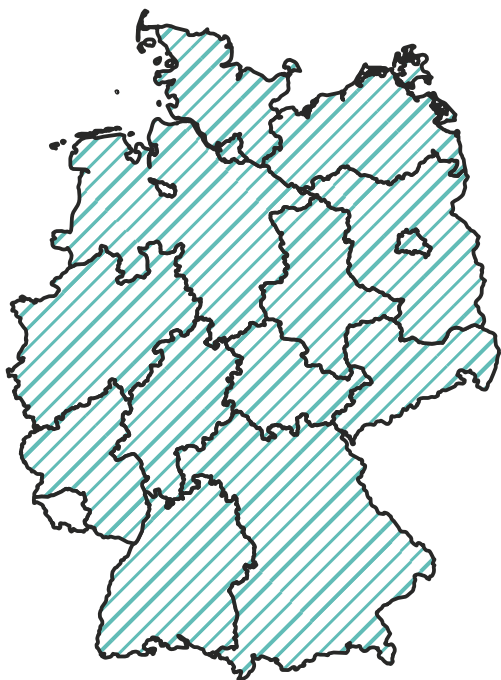

2017

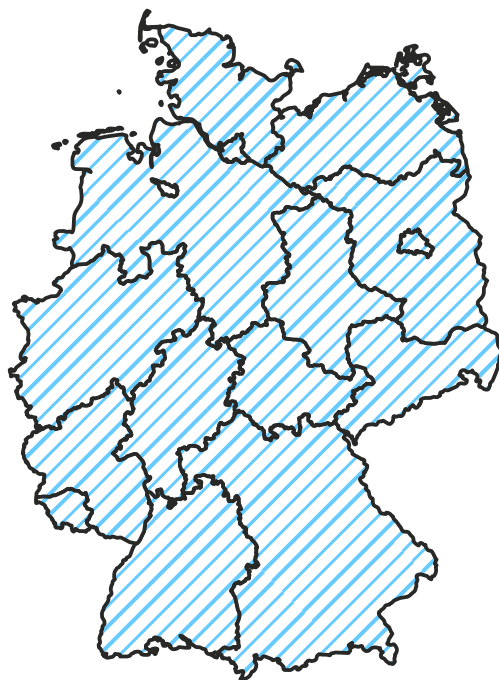

2018

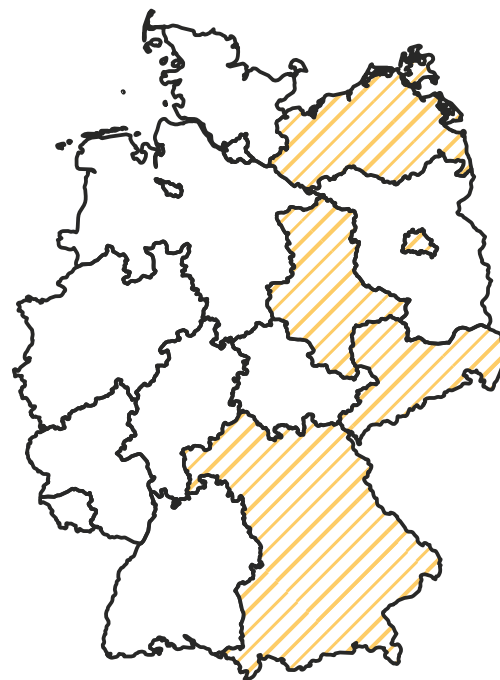

2019

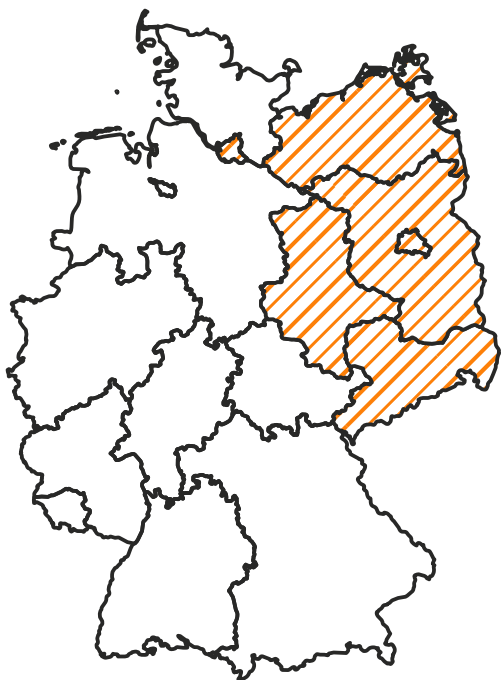

2020

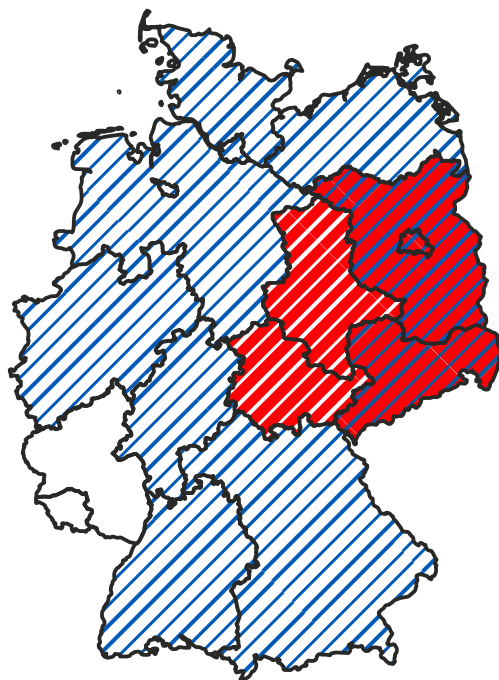

2021

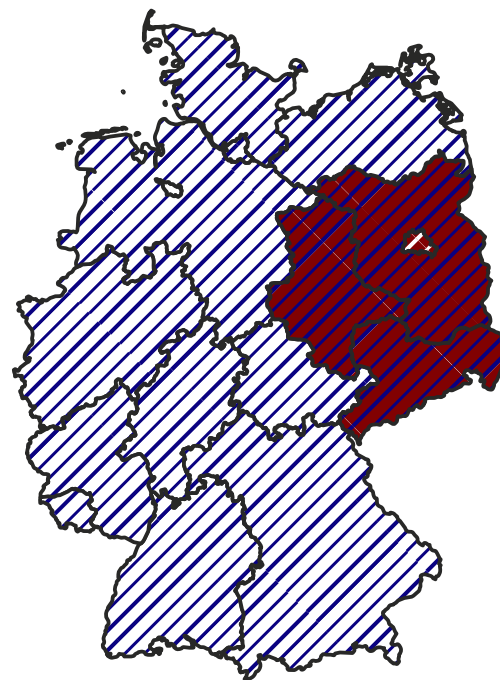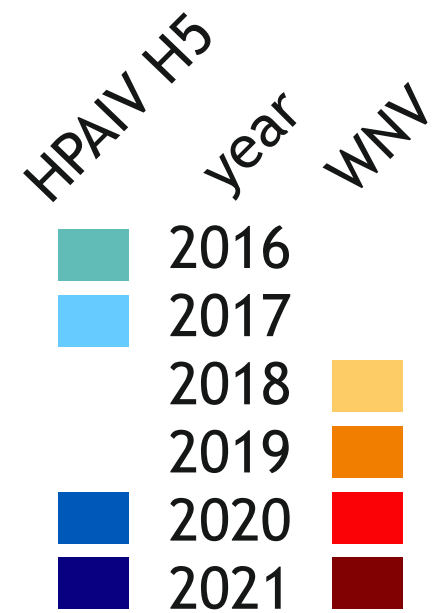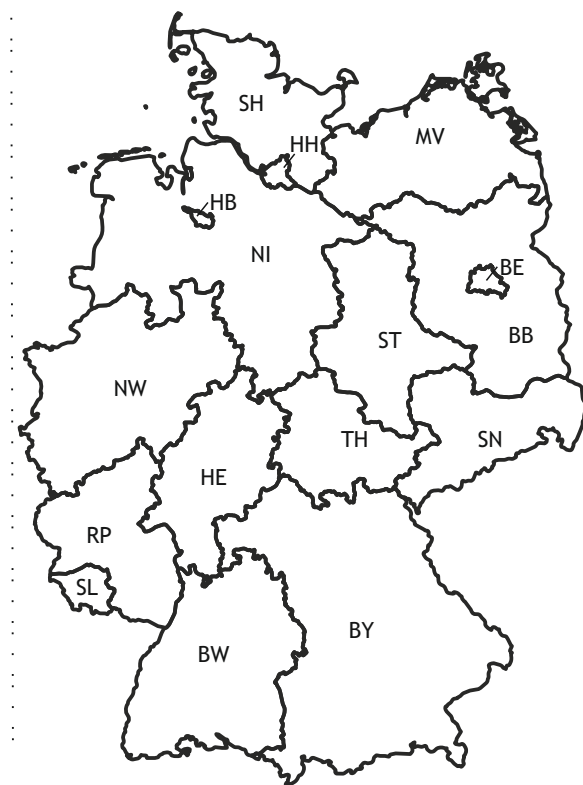

Supplement: Supplemental Material [file TEMI_A_2231561_SM6161.pdf]
